# Resistance to thyroid hormone due to a novel mutation in the thyroid beta receptor (*THRβ*) gene coexisting with autoimmune thyroid disease—A case report

**DOI:** 10.3389/fgene.2023.1051042

**Published:** 2023-04-04

**Authors:** Elżbieta Skowrońska-Jóźwiak, Agnieszka Gach, Anna Cyniak-Magierska, Anna Nykel, Monika Jurkowska, Andrzej Lewiński

**Affiliations:** ^1^ Department of Endocrinology and Metabolic Diseases, Medical University of Lodz, Lodz, Poland; ^2^ Department of Endocrinology and Metabolic Diseases, Polish Mother’s Memorial Hospital—Research Institute, Lodz, Poland; ^3^ Department of Genetics, Polish Mother’s Memorial Hospital—Research Institute, Lodz, Poland; ^4^ Genomed Health Care Center, Warsaw, Poland

**Keywords:** resistance to thyroid hormone, mutation in *THRβ* gene, thyroid autoimmunity, thyroperoxidase antibodies, thyroglobulin antibodies

## Abstract

Resistance to thyroid hormone (RTH) is a syndrome characterized by impaired responsiveness of target tissues to thyroid hormones. The relationship between RTH*β* and thyroid autoimmunity has been under research. In this study, we demonstrate a case report of a woman with a novel mutation in *THRβ* gene coexisting with autoimmune thyroid disease (AITD). The 36-year-old woman has been treated since childhood for a thyroid disease. Based on high levels of thyroid hormones (THs) and elevated concentrations of thyroperoxidase and thyroglobulin antibodies (TPOAb and TgAb, respectively), she received unnecessary long-term treatment with methimazole and finally underwent subtotal thyroidectomy. After the surgery, her TSH level remained significantly elevated, despite the treatment with 150 + 15 µg of thyroxine and triiodothyronine. A sequence analysis of the *THRβ* gene revealed a novel dinucleotide substitution affecting codon 453, resulting in the replacement of the normal proline with an asparagine (c.1357_1358delinsAA, p.(Pro453Asn)). The mutation has not been described in the literature yet; however, *THRβ* codon 453 represents a mutational hot spot, frequently altered in the TH receptor ß gene. After establishing the diagnosis of RTH, the patient was treated with 300 µg of thyroxine, which showed clinical improvement and normalization of TSH. The coexistence of RTH*β* and AITD may additionally impede establishment of a proper diagnosis, leading to unnecessary therapy and delayed correct treatment. The presented case encourages a closer cooperation between clinical endocrinologists and geneticists.

## Introduction

Resistance to thyroid hormone (RTH) is a rare syndrome characterized by impaired responsiveness of target tissues to thyroid hormones (THs), most often due to mutation in the TH receptor ß (*THRβ*) gene with an autosomal dominant inheritance pattern (Dumitrescu and Refetoff, 2013). The characteristic biochemical features of RTHß included high TH levels without TSH suppression (Pappa and Refetoff, 2021). The phenotype is variable within and between affected families. The most frequent symptoms are goiter, sinus tachycardia, learning disability, short stature, and delayed bone age (Olateju and Vanderpump, 2006; Dumitrescu and Refetoff, 2013).

In this study, we present a case report of a woman with RTH*β* due to a novel *THRβ* gene mutation coexisting with AITD.

## Case description

The patient was a 36-year-old woman with RTH*β* due to a novel *THRβ* gene mutation coexisting with AITD. After thyroid surgery, she was admitted to the Department of Endocrinology due to significantly elevated TSH, which was persistent despite the treatment with 150 µg of thyroxine and 15 µg triiodothyronine. Her history of thyroid-related problems started 25 years ago including a small goiter, palpitations, and at the same time cold intolerance and skin dryness. Clinical symptoms were accompanied by peculiar and disturbing laboratory results (elevated TH levels with normal/slightly elevated TSH). For many years, she received anti-thyroid drugs and/or different doses of thyroxine periodically; however, pharmacological treatment failed to normalize her thyroid hormone levels, and this resulted in patient’s frustration. In 2000, she was disqualified from treatment with radioiodine with suspicion of the RTH*β* syndrome. Unfortunately, genetic testing was not performed at that time. Finally, in 2003, she underwent subtotal thyroidectomy for “suspicion of Graves–Basedow disease.” Her laboratory results at that time were as follows: increased concentrations of THs accompanied by normal TSH levels [FT4: 45.7 pmol/L (N: 10–25), FT3: 22.3 pmol/L (N: 2.25–6), and TSH: 1.53 mIU/mL] and elevated levels of TgAb and TPOAb (TRAB was not available at that time). The results were obtained from patient’s documentation; however, the assay type remains unknown. Before surgery, thyroid ultrasound showed the gland of a normal size (right lobe: 19 × 16 × 57 mm, and left lobe: 19 × 15 × 50 mm); in the right lobe, two hypoechogenic lesions with the diameter of 10 mm and 6 mm were found, with no pathological lymph nodes. Fine aspiration biopsy (FNAB) of these nodules revealed the presence of benign lesions (category no. 2—according to TBSRTC classification), confirmed by histopathology. After the surgery, she chronically felt weak and sleepy. Despite the treatment with 150 μg/d of thyroxine and 15 μg/d of triiodothyronine (Euthyrox N 75 1 × 1 and Novothyral 75 1 × 1), the levels of TSH exceeded 100 mIU/L (N: 0.27–4.2), but TH levels were normal; the other results are as follows: FT3: 3.38 pg/mL (N: 2.6–4.4), FT4: 1.78 ng/dL (N: 0.93–1.7), anti-Tg: 188.7 IU/mL (N:<115), anti-TPO: 180.3 (N:<34) IU/mL, and TRAb: <0.3 IU/L (N:<1.75). All results were determined by commercially available electrochemiluminescence one-step immunoassays (ECLIA Cobas e601, Roche). She had a history of recurrent ear infections and mitral valve prolapse. She delivered twice: the first healthy daughter was born before thyroid surgery and the second daughter with the diagnosis of hypoacusis was born after the surgery. Family history revealed Hashimoto’s disease in mother and a benign non-toxic goiter in sister. The patient was admitted to the Department of Endocrinology for additional diagnostics. On admission, she presented normal mental and physical development without significant abnormalities in physical examination. Hormonal tests (TH and TSH) were repeated on different platforms, with similar results. Compliance problems, heterophilic antibody interference, and the presence of TSH-oma (normal results of MRI and normal concentrations of other pituitary hormones, alpha subunit, SHBG, and ferritin) as causes of observed abnormalities were excluded. Then, genetic testing in the patient and her family was suggested. The patient and all family members gave their informed consent to participate in this study, and the investigation was performed in accordance with the principles of the Declaration of Helsinki.

### Further investigations and results of genetic analysis

The coding region of *THRβ* gene was sequenced using the ABI 3500 Genetic Analyzer (Life Technologies, Carlsbad, CA) and analyzed using Mutation Surveyor software v.5.0.1. The alignment to reference sequence NM_000461.4 was performed.

**FIGURE 1:**
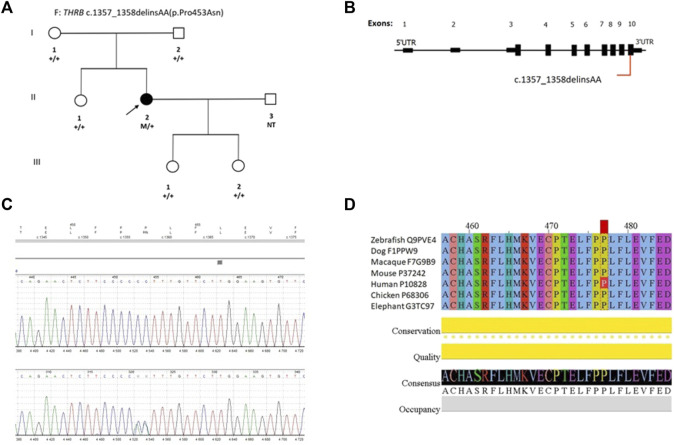
Identification of the novel *THRβ* mutation. **(A)** Pedigree of the proband harboring the novel *THRβ* mutation (p.Pro453Asn). The proband harboring *de novo* heterozygous mutation is identified by the arrow. Circles denote female members; squares denote male members. The sequencing results for the c.1357_1358delinsAA (p.Pro453Asn) mutation are noted below the corresponding individual. M, mutation; NT, not tested. **(B)** Schematic presentation of *THRβ* gene; variant’s position identified in this study is indicated in red. **(C)** Results of direct DNA sequencing for *THRβ* mutation in the proband. **(D)** UniProt alignment of the *THRβ* region containing the variant identified in this study and amino acid variations across species from zebrafish to human.

Sequence analysis revealed a novel heterozygous delins mutation c.1357_1358delinsAA in exon 10 (Figure 1). This variant is located in a highly conserved region encoding functionally important domains: nuclear hormone receptor and ligand-binding domain. The missense mutation created a substitution of proline to asparagine at codon 453 (p.Pro453Asn). This variant was not previously reported in any genetic databases (HGMD, ClinVar, ExAC, 1000 Genomes Project, and GnomAD). Moderate physicochemical differences between Pro and Asn were observed (Grantham dist.: 91 [0-215]). The variant was qualified as disease causing by SIFT (score: 0, median: 4.32) and as probably damaging by PolyPhen (score: 1). This mutation was absent in other family members.

After establishing the diagnosis of RTH, the dose of thyroxine was increased to 300 µg, which showed clinical improvement and normalization of TSH.

## Discussion

A 36-year-old woman with a novel germline mutation in *THRβ* gene accompanied by AITD is presented. The identified mutation, [c.1357_1358delinsAA (p.(Pro453Asn)] has not been reported yet, although codon 453 seems to be especially predisposed to alterations, with seven different genetic variants reported so far and around 60 families affected (Dumitrescu and Refetoff, 2013). Virtually, all pathogenic *THRβ* variants locate in binding domains of the protein, encoded by exons 7–10 (Dumitrescu and Refetoff, 2013; Pappa and Refetoff, 2021). Family testing revealed that the patient’s variant has arisen *de novo*. Such mutations are responsible for RTHß in about 20% of cases, in contrast to familial occurrence of RTH, which is frequent and has been documented in approximately 75% of cases (Olateju and Vanderpump, 2006).

Our patient experienced delayed diagnosis, which led to unnecessary and invasive treatment. According to the literature and data on diagnostic errors and as a result of failure to differentiate RTH, inappropriate therapy was delivered in one-third of the reported cases of RTH (Olateju and Vanderpump, 2006), since RTH is a rare medical condition, with various clinical presentations. RTH is most commonly mistaken as autoimmune thyroid hyperthyroidism (Olateju and Vanderpump, 2006; Pappa and Refetoff, 2021). In addition to RTH, our patient demonstrated biochemical features of AITD (high titers anti-TPO and anti-Tg antibodies), which additionally impeded establishment of a proper diagnosis. Thyroid autoimmunity is characterized by the high prevalence in general population; therefore, coexistence of RTH with AITD is considered to be coincidental (Larsen et al., 2013). However, a causal relationship between RTH and AITD has been also suggested (Tomer and Davies, 2003; Gavin et al., 2008; Barkoff et al., 2010; Wu et al., 2018). In the study of 330 individuals with RTHß and 92 unaffected first-degree relatives, two-fold higher frequency of positive thyroid auto-antibodies was shown (Barkoff et al., 2010). Surprisingly, the risk of AITD was higher in men with RTH than in women, in contrast to general population (Barkoff et al., 2010). Susceptibility to AITD is related to an underlying genetic disposition (Tomer and Davies, 2003). Our subject had maternal history of Hashimoto’s disease. One of the hypotheses, affecting the association between AITD and RTH, assumes that chronically elevated TH concentrations in RTH may stimulate the immune system through receptor alpha (Barkoff et al., 2010). Another proposed pathophysiologic mechanism suggests the influence of chronic TSH elevation which stimulates lymphocytes to produce the pro-inflammatory cytokine TNF-alpha (Gavin et al., 2008).

Optimization of thyroxine dose in replacement therapy was challenging. It is consistent with the literature data that since patients who have previously been misdiagnosed and treated with ablative therapy resulted in thyroid dysfunction, they usually need supraphysiological dose of thyroid hormone (Ingbar et al., 1982; Olateju and Vanderpump, 2006). The aim of the treatment is to achieve the lowest TSH level, which is not accompanied by tachycardia. Another approach is to maintain TSH at levels lower than the mean TSH level of the affected family members (Tran, 2006).

Identification of the *THRβ* pathogenic variant in our patient not only confirmed the proper diagnosis but also changed management, enabling dosing of thyroxine in supraphysiological doses. The presented case encourages a better cooperation between endocrinologists and geneticists and earlier intervention of genetic diagnostics, which can eliminate unnecessary procedures and treatment in RTH patients.

## Data Availability

The data presented in the study are deposited in the ClinVar repository, accession number VCV002442325.1.
